# Enalapril overcomes chemoresistance and potentiates antitumor efficacy of 5-FU in colorectal cancer by suppressing proliferation, angiogenesis, and NF-κB/STAT3-regulated proteins

**DOI:** 10.1038/s41419-020-2675-x

**Published:** 2020-06-24

**Authors:** Yushan Yang, Lulu Ma, Yiming Xu, Yun Liu, Wenya Li, Jianchun Cai, Yiyao Zhang

**Affiliations:** 1 Department of Gastrointestinal Surgery, Zhongshan Hospital of Xiamen University, Xiamen, 361000 China; 2 Gastrointestinal Oncology Center of Xiamen University, Xiamen, 361000 China; 3 Medical College of Xiamen University, Xiamen, 361000 China; 4 General Surgery, Zhongshan Hospital of Xiamen University, Xiamen, 361000 China

**Keywords:** Cancer therapeutic resistance, Translational research

## Abstract

5-Fluorouracil (5-FU) is one of the most effective drugs for the treatment of colorectal cancer (CRC). However, there is an urgent need in reducing its systemic side effects and chemoresistance to make 5-FU-based chemotherapy more effective and less toxic in the treatment of CRC. Here, enalapril, a clinically widely used antihypertensive and anti-heart failure drug, has been verified as a chemosensitizer that extremely improves the sensitivity of CRC cells to 5-FU. Enalapril greatly augmented the cytotoxicity of 5-FU on the cell growth in both established and primary CRC cells. The combination of enalapril and 5-FU synergistically suppressed the cell migration and invasion in both 5-FU-sensitive and -resistant CRC cells in vitro, and inhibited angiogenesis, tumor growth, and metastasis of 5-FU-resistant CRC cells in vivo without increased systemic toxicity at concentrations that were ineffective as individual agents. Furthermore, combined treatment cooperatively inhibited NF-κB/STAT3 signaling pathway and subsequently reduced the expression levels of NF-κB/STAT3-regulated proteins (c-Myc, Cyclin D1, MMP-9, MMP-2, VEGF, Bcl-2, and XIAP) in vitro and in vivo. This study provides the first evidence that enalapril greatly sensitized CRC cells to 5-FU at clinically achievable concentrations without additional toxicity and the synergistic effect may be mainly by cooperatively suppressing proliferation, angiogenesis, and NF-κB/STAT3-regulated proteins.

## Introduction

The incidence and mortality of colorectal cancer (CRC) are in the forefront of malignancies^1^. Most patients are usually diagnosed at an advanced stage and chemotherapy-based comprehensive treatment is the best choice for CRC^2^. However, the toxic side effects and therapeutic resistance of chemotherapy drugs are the main obstacles to successfully cure CRC^2,3^.

5-Fluorouracil (5-FU) is one of the most widely used chemotherapy drugs in CRC with an overall response rate of only 10–15% due to chemoresistance^4^. 5-FU-based chemotherapy (such as XELOX and FOLFOX) is still the current first-line chemotherapeutic drug for advanced CRC regardless of the emergence of new chemotherapy drugs. However, chemoresistance and severe side effects have become the major hurdle to improve combined treatment efficacy^2,5,6^. To overcome chemoresistance and reduce side effects, a number of drugs are tested in clinical trials, but no significant progress has been made so far, calling for more efforts to identify new drugs that overcome the resistance of 5-FU with less toxicity to improve outcomes of patients with CRC.

Angiotensin-converting enzyme (ACE) inhibitors are widely used antihypertensive and anti-heart failure drugs clinically clinically^7^. ACE converts angiotensin I to angiotensin II, which is one of the most important angiogenic factors in activating nuclear factor-κB (NF-κB) signaling to upregulate vascular endothelial growth factor (VEGF) expression^8,9^. Epidemiological data have suggested that long-term use of ACE inhibitors may decrease the risk of cancer in high-pressure patients, although these results were contradictory^10,11^. ACE inhibitors including enalapril also have been confirmed to suppress tumorigenesis and angiogenesis in other cancer mouse models, and that suppression of the VEGF level may be involved in the mechanism of this inhibitory effect^8,12,13^. VEGF is considered to be one of the most critical angiogenic factors through promoting the formation of new blood and lymphatic vessels^14^, which is a critical process in tumor growth and metastasis^15^. It has been also reported that enalapril reduces the expression of NF-κB in pancreas cancer^13^. However, the underlying mechanisms of the antitumor effects of ACE inhibitors are still unknown.

NF-κB/signal transducer and activator of transcription 3 (STAT3) signaling is overactivated in CRC and their regulated proteins, such as VEGF, matrix metalloproteinase (MMP)-9 and MMP-2, c-Myc and Cyclin D1, and Bcl-2 and X-linked inhibitor of apoptosis protein (XIAP), have been linked with tumor angiogenesis, invasion, metastasis, pro-proliferation, and anti-apoptosis, respectively^16–20^. It has been reported that activation of the NF-κB/STAT3 signaling pathway is one of the critical mechanisms for 5-FU resistance^16,21,22^. In addition, it is worth noting that 5-FU treatment stimulates VEGF-mediated angiogenesis^23,24^, so a drug can inactivate the aberrant activation of NF-κB/STAT3 and VEGF would possibly overcome chemoresistance and potentiate the efficacy of 5-FU in CRC. ACE inhibitor captopril has been shown to increase the sensitivity of drug-resistant breast cancer cells to tamoxifen in a mouse model^25^, which is consistent with the result from the clinical retrospective analysis that the combination of ACE inhibitors with standard chemotherapeutic drugs may improve the therapeutic effects in pancreatic and lung cancer^26,27^. However, similar effects of ACE inhibitors in CRC has not been tested yet.

Therefore, the purpose of this study is to evaluate the possibility of enalapril serving as a sensitizer to reverse 5-FU resistance and potentiate the antitumor effects of 5-FU against CRC, and to explore the side effects when combining the two agents and potential synergistic antitumor mechanisms. To our knowledge, the present study is the first to evaluate the role of enalapril in overcoming 5-FU resistance in CRC.

## Results

### Enalapril increases the chemosensitivity of CRC cells to 5-FU in vitro

**Fig. 1:**
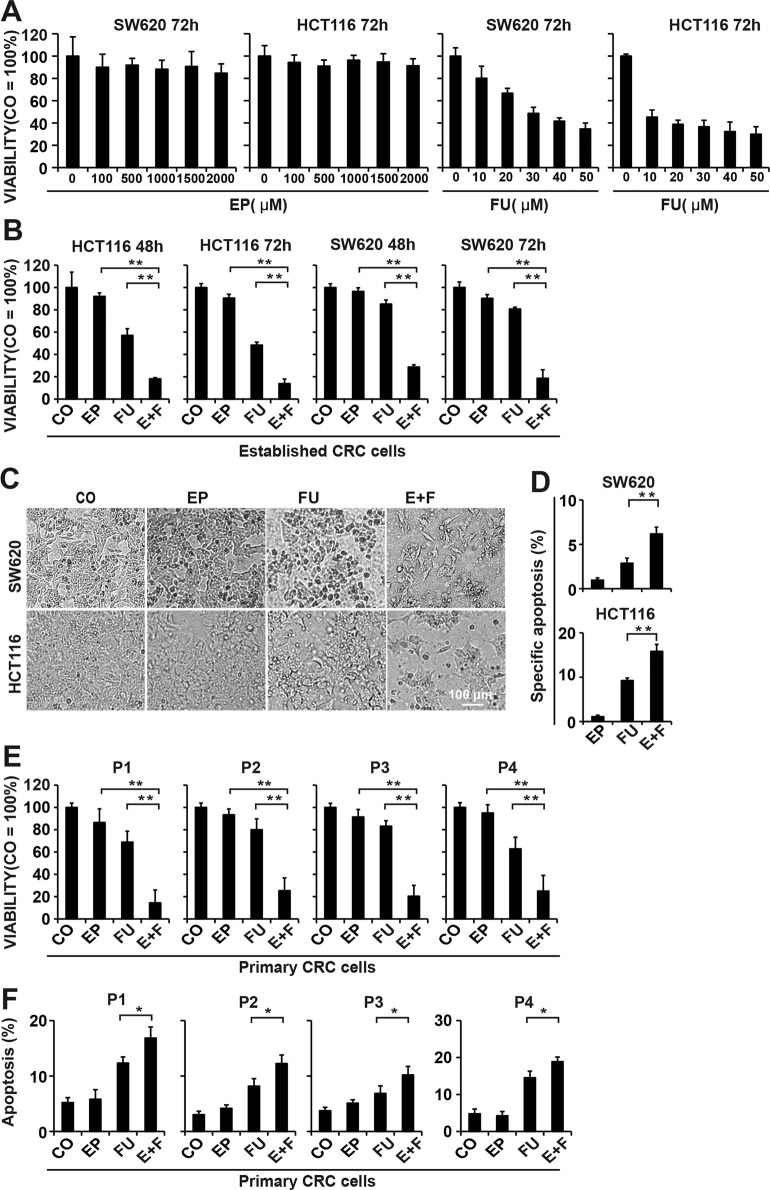
Enalapril overcomes 5-FU resistance and potentiates the proliferation-inhibitory effects of 5-FU in CRC cells. **a** HCT116 and SW620 cells were treated with increasing concentrations of enalapril (EP) or 5-FU (FU) for 72 h, and the cell viability was measured by MTT assay. **b** HCT116 and SW620 cells were treated with EP (100 μM), FU (10 μM), or the two agents combined (E+F) for 72 h, and the cell viability was measured by MTT. **c** The representative morphology of SW620 cells after 72 h treatment was shown by microscopy at ×200 magnification. **d** HCT116 and SW620 cells were treated as described in **b** for 48 h and the cell apoapsis was analyzed by flow cytometry. **e** The primary CRC cell lines P1, P2, P3, and P4 were treated as described above, and the cell viability was measured by MTT. **f** The primary CRC cells were treated as described above and, after 48 h, the cell apoapsis was measured by flow cytometry. The data are presented as means ± SD from three separate experiments (*n* = 8 per group). Statistical analysis performed using two-tailed *t*-test (**P < 0.01, *P < 0.05).

We examined the effect of enalapril and 5-FU on the viability of the SW620 and HCT116 cells with MTT (3-(4, 5-dimethylthiazol-2-yl)-2, 5-diphenyltetrazolium bromide) assay. Treatment with enalapril had a very limited effect on the viability of both CRC cell lines even at a high concentration (100–2000 μM) for 72 h (Fig. 1a and Supplementary Fig. 1A). Therefore, the lowest dose of enalapril 100 μM was sorted out for further combination studies. 5-FU treatment reduced the viability of HCT116 and SW620 cells in a time- and dose-dependent manner with increasing 5-FU concentrations (10–50 μM), and HCT116 cells were susceptible to 5-FU treatment according to the 50% inhibitory concentration (Fig. 1a and Supplementary Fig. 1B). 5-FU (10 μM) was selected for further combinative studies due to its weak cytotoxicity in SW620 cells.

Enalapril did not significantly affect the viability of SW620 and HCT116 cells; in contrast to enalapril, 5-FU reduced the viability of SW620 cells by 20% and HCT116 cells by 51% at a concentration of 10 μM. However, enalapril combination with 5-FU extremely decreased cell viability in HCT116 cells by 87% and in SW620 cells by 83% at 72 h, which was much stronger than each agent alone in a time-dependent manner (Fig. 1b, c and Supplementary Fig. 1C), suggesting that the different susceptibility of 5-FU in both cell lines disappeared after the addition of enalapril in both moderately sensitive and resistant CRC cells.

Next, we analyzed the cell apoptosis by flow cytometry and found that treatment with 5-FU alone for 48 h only induced limited apoptosis in both cell lines, and HCT116 cells showed a little higher sensitivity to 5-FU-induced apoptosis than SW620 cells. Although enalapril had no significant effect on cell apoptosis, upon the combination with 5-FU still produced more profound apoptosis than 5-FU alone. However, the specific apoptotic rate was no more than 17% even in the combination group (Fig. 1d and Supplementary Fig. 2A). The results of cell cycle distribution evaluated by flow cytometry also showed that most colon cancer cells in the combined treatment group were in G0-G1 phase (Supplementary Fig. 2B). Hence, the growth-inhibitory effect of combination treatment on CRC cells was mainly by inhibiting cell proliferation rather than inducing cell apoptosis.

To further confirm the synergistic growth-inhibitory effect between enalapril and 5-FU more clinically relevant, we detected the effect in the primary human CRC cell lines P1 (median 5-FU-resistant), P2 and P3 (5-FU-resistant), and P4 (median 5-FU-resistant). Similarly, both enalapril and 5-FU only had a weak effect on cell growth in four cell lines. However, 5-FU combined with enalapril extremely suppressed the growth of all primary CRC cell lines mainly due to inhibit cell proliferation (Fig. 1e, f).

These results indicated that enalapril extremely enhanced the chemosensitivity of CRC cells to 5-FU mainly through inhibiting cell proliferation.

### Enalapril enhances the antitumor, antiproliferative, and antiangiogenic efficacy of 5-FU in vivo

**Fig. 2:**
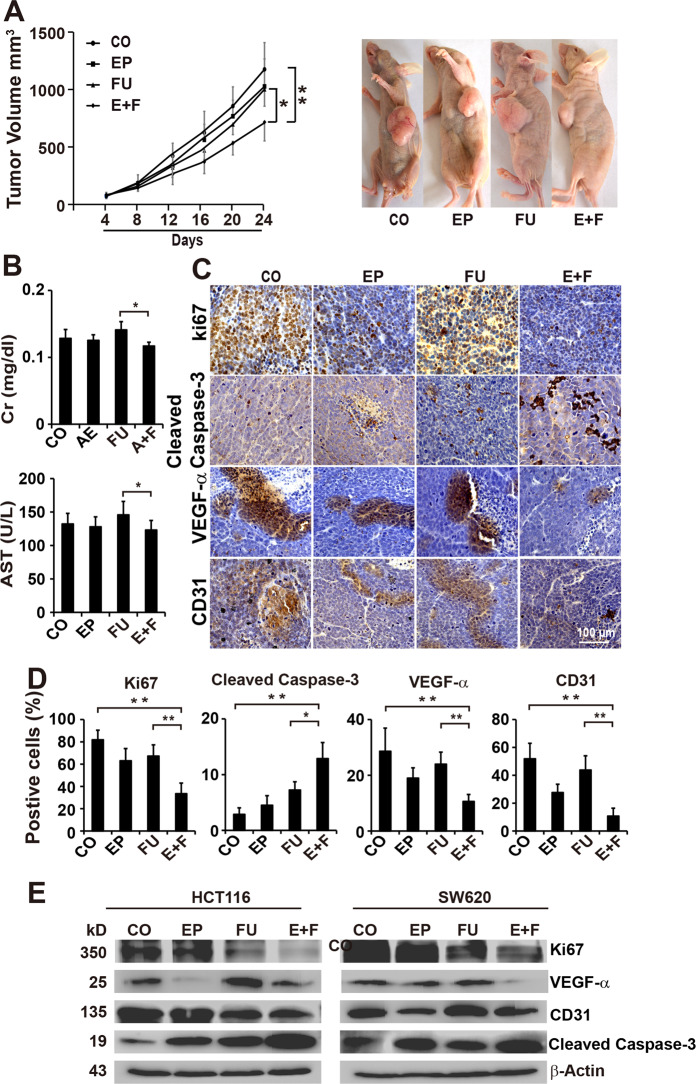
Combination of enalapril and 5-FU synergistically inhibits tumor growth, cell proliferation, and angiogenesis in vivo. **a** SW620 cells were injected subcutaneously into the left flanks of nude mice. Twenty-four mice were allocated randomly to the control group (100 μl, p.o., daily), EP group (0.6 mg/kg, p.o., daily), FU group (30 mg/kg, i.p., 2 times/week), and E+F. The xenografts were resected on day 24 and the tumor volumes were measured and analyzed. Representative images of mice bearing tumor xenografts for each group were shown on the right (*n* = 6 per group). **b** Blood from mice was collected at the end of the experiment and the levels of plasma creatinine (Cr) and the levels of aspartate aminotransferase (AST) were analyzed. **c** Immunohistochemical analysis of CD31, VEGF-α, Ki67, and Caspase-3 in each treatment xenograft tumor tissues. **d** The number of positive cells was quantified in ten visual fields at ×400 magnification and the means ± SD are shown in the diagrams (n = 10 per group). **e** Western blot analysis of the expression of CD31, VEGF-α, Ki67, and Caspase-3 in SW620 cells treated with EP, FU, or E+F. β-Actin served as a control. The data are presented as means ± SD from three separate experiments (**c**–**e**). Statistical analysis performed using two-tailed *t*-test (**P < 0.01, *P < 0.05).

To confirm our in vitro results, we assessed the in vivo effect of 5-FU plus enalapril on the growth of CRC. In SW620 xenografts, the final tumor volume was smaller in the enalapril or 5-FU group compared with the control group, but it did not reach statistical difference. However, The final tumor volume in the combination treatment group was significantly smaller compared with the control or 5-FU group (Fig. 2a). Tumor weight also showed that cotreatment with 5-FU and Enalapril significantly reduced tumor weight with no undesirable effects of the cotreatment on mice behavior and body weight (Supplementary Fig. 2A, B). Accordingly, results from liver and kidney function tests also showed that serum creatinine, blood urea nitrogen, alanine aminotransferase, and aspartate aminotransferase in combination treatment represented a downward trend as compared with 5-FU treatment (Fig. 2b and Supplementary Fig. 2C, D), suggesting the safety of the combinative treatment with no extra in vivo toxicity added.

To investigate the potential mechanisms for tumor growth inhibition, we next examined the expression cleaved caspase-3, a hallmark of apoptosis, and Ki67, a hallmark of proliferation in SW620 xenograft tissues by immunohistochemistry. Enalapril in combination with 5-FU almost completely abolished the expression of Ki67 and slightly upregulated the expression of cleaved caspases-3, whereas the single treatment had no or only limited effect (Fig. 2c, d). It reported that ACE inhibitor perindopril-inhibited tumor growth was associated with the suppression of angiogenesis^12^. To investigate whether the effect of enalapril on tumor development was accompanied by suppression of tumor angiogenesis, the microvessel density marker CD31 and VEGF-α were examined in tumor tissues by immunohistochemistry. The results showed that enalapril alone significantly suppressed the expression of CD31 and VEGF-α, and the presence of 5-FU further enhanced the downregulation of CD31 and VEGF-α (Fig. 2c, d). Accordingly, the western blotting results showed that single treatment was minimally effective and 5-FU even induced the expression of VEGF-α. However, the combination of the two effectively inhibited the expression of Ki67, CD31, and VEGF-α, and upregulated the expression of cleaved caspase-3 in W620 cells (Fig. 2e).

Taken together, these data indicated that enalapril enhanced the antitumor effects of 5-FU in chemotherapy-resistant tumor xenografts and was associated with antiangiogenic, antiproliferative, and pro-apoptotic mechanisms.

### Enalapril augments the effects of 5-FU in suppressing the CRC migration, invasion, and metastasis

**Fig. 3:**
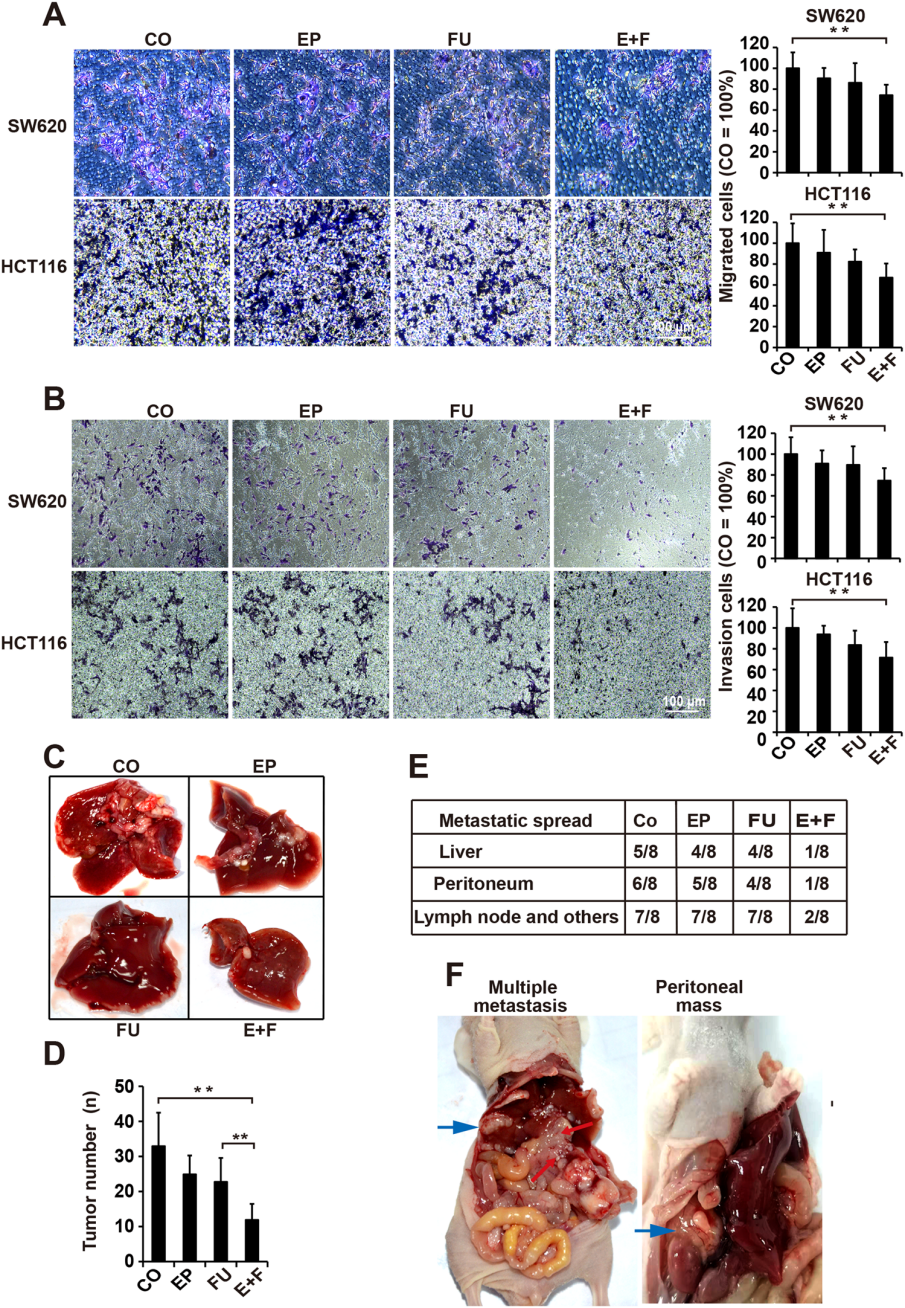
Enalapril augments the effects of 5-FU against tumor cells migration, invasion, and metastasis in CRC. **a** The migration capacities of SW620 and HCT116 cells were determined using the Transwell chambers after 24 h of treatment with 100 μM EP, 10 μM FU, or E+F (*n* = 5 per group). Representative photographs of the migrated cells were presented. The number of migrated cells was quantified in ten visual fields at ×200 magnification and the means ± SD are shown in the diagrams. The number of migratory cells in the control is set to 100%. **b** The invasion capacities of SW620 and HCT116 cells were detected using the Transwell chambers coated with Matrigel after 24 h of treatment as described above (*n* = 5 per group). The number of migrated cells was quantified in ten visual fields at ×200 magnification. **c** SW620 cells were injected into the splenic vein of mice and the liver metastasis was assessed after treatment with EP, FU, or E+F (*n* = 5) for 7 weeks. Representative photograph of the metastatic nodules in the liver was shown and **d** the number of liver metastatic nodules was counted and plotted. **e** SW620 cells were orthotopically injected to observe the metastatic spread in vivo after treatment as described above (*n* = 8 per group). Mice were killed after 8 weeks. **f** Representative photographs of the metastasis were shown. The data are presented as means ± SD from three separate experiments (**a**, **b**). Statistical analysis (**a**, **b**, and **c**) performed using two-tailed *t*-test and Mann–Whitney test (**d**, **e**) (**P < 0.01, *P < 0.05).

We next tested the migratory and invasive abilities of SW620 and HCT116 cells after each treatment by Transwell assays. The number of migrated and invasive cells was not significantly decreased in single treatment, whereas the combination group was found to be the most effective in reducing migrated and invasive cells compared with single treatment (Fig. 3a, b). CRC often develops liver and peritoneum metastasis, so we evaluated whether enalapril could enhance the effect of 5-FU on CRC metastasis in vivo. First, we established a liver metastasis model of nude mice by portal vein injection of SW620 cells. Neither enalapril nor 5-Fu alone showed a significant anti-metastatic efficacy on the liver, whereas the combination of enalapril and 5-FU dramatically diminished the tumor metastasis as indicated by the number of metastatic nodules in the liver (Fig. 3c, d). To further confirm these results, a model of peritoneal metastasis was established by injecting SW620 cells into nude mice. The results showed that liver metastasis occurred in four out of eight animals treated with 5-FU and enalapril alone, whereas only one out of eight were found in combination-treated animals (Fig. 3e). Besides, combined treatment can greatly reduce the lymph node, peritoneal metastasis, and other metastasis, but the monotherapy adopting 5-FU or enalapril alone was ineffective (Fig. 3e, f), suggesting a synergistic effect between 5-FU and enalapril in suppressing CRC metastasis.

Collectively, these results demonstrated that enalapril and 5-FU synergistically inhibited the migration, invasion, and metastasis of CRC.

### Enalapril and 5-FU synergistic suppresses EMT signaling in CRC

**Fig. 4:**
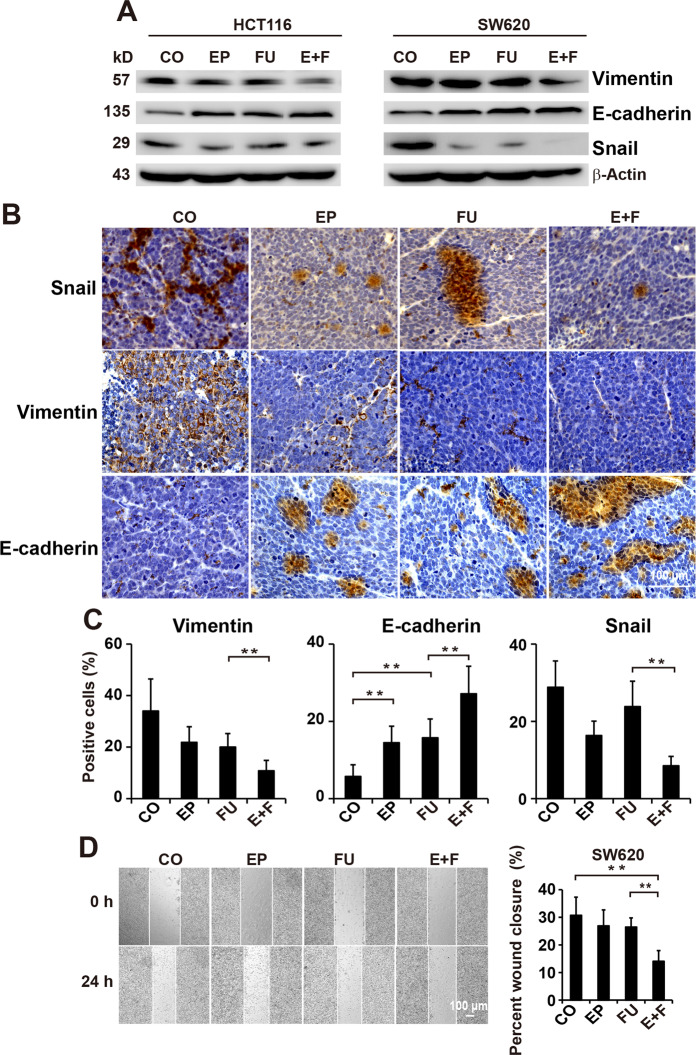
Enalapril enhances the effects of 5-FU in suppressing EMT signaling in CRC. **a** Western blot analysis of vimentin, E-cadherin, and Snail in CRC cells treated with EP, FU, or E+F. **b** Immunohistochemical analysis of Vimentin, E-cadherin, and Snail in tumor tissue samples treated with EP, FU, or E+F (*n* = 5 per group). **c** The number of positive cells was quantified in ten visual fields at ×400 magnification and the means ± SD are shown in the diagrams. **d** SW620 cells were scratched with a pipette tip and then treated with EP, FU, or E+F (*n* = 6 per group). The images were taken 0 and 24 h after wound formation and the percent of wound closure was analyzed. The data are presented as means ± SD from three separate experiments. Statistical analysis performed using two-tailed *t*-test (**P < 0.01, *P < 0.05).

EMT is closely related to increased chemoresistance and tumor metastasis^28^. To address this issue, western blotting was employed to verify whether enalapril can enhance the effect of 5-FU on the EMT-associated key regulatory molecules, such as Vimentin, E-cadherin, and Snail. As showed in Fig. 4a, the expression of Vimentin was significantly inhibited, whereas the expression of E-cadherin was markedly increased in SW620 and HCT116 cells in the combination group compared with the control or 5-FU group. Although 5-FU treatment induced the expression of Snail with no effect on the expression of vimentin, the cotreatment with enalapril was proved to be the strongest to downregulate vimentin and Snail, and upregulate E-cadherin in HCT116 and SW620, indicating the synergistic effect between enalapril and 5-FU in inhibiting EMT process. To confirm the above results, these protein levels were evaluated in SW620-derived xenograft tumors by immunohistochemical analysis. Similarly, the protein level of E-cadherin was remarkedly increased and the protein levels of Snail and Vimentin were significantly decreased in combination-treated tumors compared with 5-FU-treated tumors (Fig. 4b, c). As EMT is associated with enhanced cell migration, we evaluated whether the treatment affects the cell motility of chemoresistant SW620 cells. Neither 5-FU nor enalapril could inhibit the migration of CRC cells, whereas 5-FU strongly prevented the cell migration on coincubation with enalapril (Fig. 4d).

These data provided evidence that the combination of enalapril and 5-FU synergistically suppressed the EMT process in CRC.

### Enalapril potentiates the effect of 5-FU in downregulating the expression of NF-κB/STAT3-regulated proteins

**Fig. 5:**
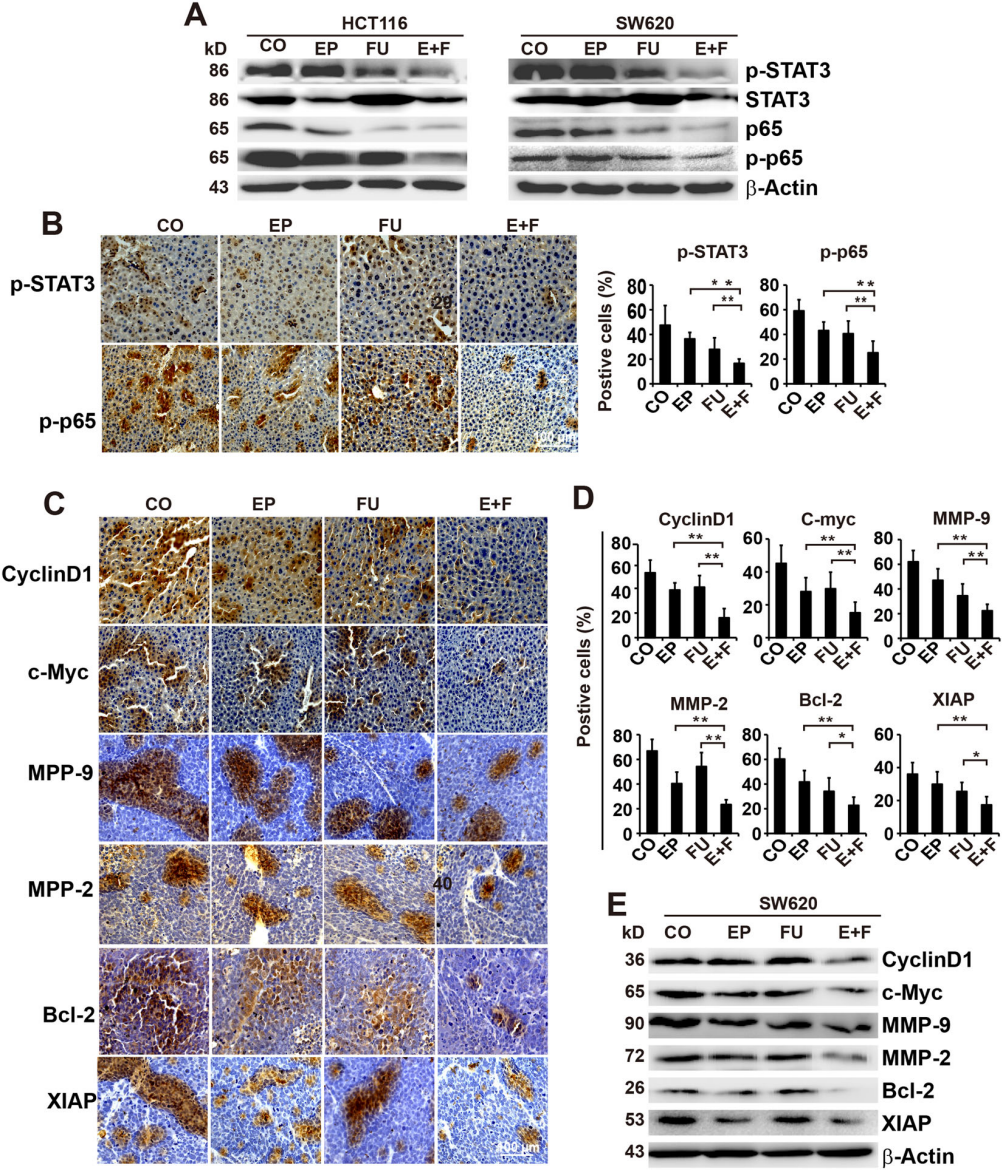
Enalapril enhances the efficacy of 5-FU in suppressing the expression of NF-κB/STAT3 and NF-κB/STAT3-regulated proteins in CRC. **a** SW620 and HCT116 cell lysates were analyzed for p-STAT3, STAT3, p-P65, and P65 by western blotting. **b** Immunohistochemical analysis of p-STAT3 and p-P65 in tumor tissue samples treated with EP, FU, or E+F (*n* = 5 per group). The number of positive cells was quantified in ten visual fields at ×400 magnification and the means ± SD are shown in the diagrams. **c** Representative pictures and immunohistochemical analysis for the expression of STAT3-regulated proteins Cyclin D1, c-Myc, MPP-9, MMP-2, Bcl-2, and XIAP in tumor tissues (*n* = 5 per group). **d** The number of positive cells was quantified in ten visual fields at ×400 magnification and the means ± SD are shown in the diagrams. **e** Western blot analysis for the expression of NF-κB/STAT3-regulated proteins Cyclin D1, c-Myc, MPP-9, MMP-2, Bcl-2, and XIAP in SW620 cells. The data are presented as means ± SD from three separate experiments. Statistical analysis performed using two-tailed *t*-test (**P < 0.01, *P < 0.05).

NF-κB/STAT3 signaling is thought to play an important role in tumor proliferation, progression, and chemoresistance in CRC^18^. To assess the mechanism responsible for these results, we examined the expression of STAT3 and p65 by western blot analysis. The expression of p-STAT3 and p-p65 was slightly inhibited by 5-FU and completely suppressed upon combination treatment with enalapril in both CRC cell lines (Fig. 5a). Accordingly, we observed significantly decreased expression of STAT3 and p65 in tumors from the combination treatment group compared with those from the enalapril or 5-FU group (Fig. 5b and Supplementary Fig. 5A, B).

Next, we evaluated the expression of the NF-κB/STAT3-regulated downstream proteins Cyclin D1 and c-Myc, which are involved in tumor cell proliferation, Bcl-2 and XIAP, whose overexpression is closely related to tumor survival and chemoresistance, and MMP-9 and MMP-2 that are associated with tumor invasion and metastasis. Immunohistochemical analysis showed that enalapril in combination with 5-FU significantly decreased the expression of all of these proteins compared with single-agent treatment in CRC tumor tissues (Fig. 5c, d), which were further confirmed by our western blotting results that combinative treatment strongly suppressed the expression of these proteins compared with single treatment in SW620 cells (Fig. 5e).

These data suggested enalapril combined with 5-FU could synergistically inhibit the expression of STAT3 and STAT3-regulated genes.

## Discussion

At present, the most commonly used method to prevent chemoresistance and improve chemotherapy efficacy is the combination of two or three or even four drugs^29^. Although the combinational use of 5-FU with other agents, such as FOLFOX and FOLFIRI, has improved the progress of CRC to a certain extent, it meanwhile brings more significant toxic side effects^29,30^. Therefore, how to improve the efficacy of 5-FU without additional side effects caused by these drugs has become an urgent problem to be solved. In the present study, we for the first time demonstrated that enalapril, a clinically widely used antihypertensive drug, significantly enhanced the efficacy of 5-FU in suppressing tumor growth and metastasis in CRC without additional toxicity through inhibition of proliferation, angiogenesis, and NF-κB/STAT3-regulated proteins, showing an attractive therapeutic strategy for CRC.

Tumor growth and metastasis depend on the formation of tumor blood vessels. Furthermore, tumor blood vessels are often disorganized that prevent the delivery of chemotherapeutic drugs to tumor tissues, thus leading to chemoresistance. Therefore, the current clinical strategy for the treatment of metastatic CRC is to target both tumor growth and angiogenesis. Clinical studies have been shown that antiangiogenic drug bevacizumab, a VEGF inhibitor, in combination with 5-FU synergistically improve the outcomes of metastatic CRC^31,32^. However, bevacizumab is not only expensive but also often has some serious side effects including life-threatening hemorrhage and venous thromboembolism^33^. Therefore, a cheap agent that can inhibit tumor angiogenesis and enhance the efficacy of 5-FU without unwanted side effects will have an important clinical value. Our results suggest that enalapril has no direct effect against the proliferation of CRC cells in vitro, but would potentially suppress tumor growth by inhibiting angiogenesis in vivo, which is consistent with the results reported previously in which ACE inhibitors including enalapril inhibited tumor development possibly by suppressing angiogenesis^12,13^. It is worth noting that the lower concentration of 5-FU can even enhance the expression of VEGF-α and promote the growth of tumor blood vessels^23,24^. Similarly, we also found it is enalapril rather than 5-FU significantly suppressed the expression of angiogenesis maker VEGF-α and CD31. However, the combinational use of 5-FU with enalapril almost completely abolished these protein expressions. Although enalapril did not affect the proliferation of CRC cells in vitro and 5-FU was ineffective in suppressing the CRC growth, bombinated treatment with enalapril and 5-FU significantly decreased vessel formation, suppressed the tumor growth and metastasis at a clinically comparable dose without additional side effects in vivo. These results proved that enalapril as an effective 5-FU sensitizer at least partly by targeting the tumor angiogenesis in CRC.

Our results demonstrated enalapril represented very limited effects on the proliferation of CRC cells even at a high concentration, whereas enalapril extremely enhanced the chemosensitivity of CRC cells to 5-FU in vitro. These data were further supported by in vivo results showing that 5-FU or enalapril alone did not significantly inhibit tumor growth and metastasis. Nevertheless, concomitant treatment with enalapril restored 5-FU sensitivity and significantly suppressed tumor growth and metastasis. These results cannot be explained solely by the inhibitory effects against tumor angiogenesis, as tumor cell proliferation is independent of angiogenesis in vitro, leading us to further explore the mechanism by which enalapril potentiates the therapeutic effects of 5-FU on CRC. As NF-κB/STAT3 is overactivated in tumors and directly or indirectly controls tumorigenesis, tumor cell proliferation and survival, metastasis, and chemoresistance, and blocking the NF-κB/STAT3 signaling has been proven to restore sensitivity towards 5-FU in CRC^18,34–37^, we assume that the above results may be due to the synergistic effect between enalapril and 5-FU in inhibiting NF-κB/STAT3 activity. Indeed, we demonstrated that neither enalapril or 5-FU had a minimal effect on inhibiting the NF-κB/STAT3 pathway; however, the combinational use of enalapril and 5-FU greatly suppressed NF-κB/STAT3. Consistent with the above results, we also found that several NF-κB/STAT3-regulated anti-apoptotic proteins, Bcl-2 and XIAP, and antiproliferative proteins, c-Myc and Cyclin D1, were only significantly suppressed by the concomitant treatment. These results indicated that enalapril enhanced the antitumor effects of 5-FU by inhibiting NF-κB/STAT3 and NF-κB/STAT3-regulated anti-apoptotic as well as antiproliferative proteins. Our results also showed that 5-FU and enalapril together strongly inhibited the expression of proliferation marker Ki67 and slightly induced the expression of pro-apoptotic protein caspase-3. Therefore, it is very likely that the synergistic suppression of NF-κB/STAT3 signaling by enalapril and 5-FU cotreatment sensitized the CRC cells to 5-FU.

Our results suggested that enalapril or 5-FU alone could not significantly inhibit the invasion and metastasis of CRC, whereas two drugs together synergistically inhibited the above effects. As tumor invasion, metastasis, and therapeutic resistance are closely related to EMT^38,39^ and NF-κB/STAT3 signaling is essential for promoting EMT in CRC^19,38,40–42^, we evaluated the effect of the combined use of the two drugs on EMT. We found that cotreatment synergistically suppressed the EMT by upregulating the epithelial cell marker E-cadherin and downregulating the mesenchymal cell markers Vimentin and Snail. In addition, we also found that enalapril and 5-FU together significantly inhibited the expression of MMP-2 and MMP-9 in vivo, which are also associated with tumor invasion and metastasis, and are regulated by NF-κB/STAT3 pathway^19^. Therefore, inhibition of EMT process is considered to be another possible mechanism by which enalapril potentiated the antitumor of 5-FU in CRC.

The concentrations of enalapril and 5-FU administered in mice were also relevant for the human body. The dose of 5-FU with 30 mg/kg was given twice a week and enalapril was given by a dose of 0.6 mg/kg daily in this study based on the previous studies^13,43^. The occurrence of severe toxicity including life-threatening cardiotoxicity caused by 5-FU-based chemotherapy is 10% to 40%, which is the main limitation for its clinical application^3^. This study showed enalapril, a clinically widely used drug for the treatment of heart failure and antihypertension, strongly enhanced the anti-colon cancer effect of 5-FU and could be applied to reduce the toxicity on tumor-bearing mice treated with 5-FU, which is consistent with previous clinical studies that enalapril was very well tolerated and safe in human subjects with protective effects on patients with renal insufficiency and against myocardial toxicity induced by chemotherapy drugs including 5-FU^7,44,45^. Therefore, the application of enalapril and 5-FU together also provides a low-toxic maintenance therapy for CRC patients after high-intensity and high-toxicity standard FOLFOX and FOLFIRI therapy. As CRC patients tend to be elderly, who often have already suffered from cardiovascular diseases, enalapril, in this case, can provide many advantages such as low price, good tolerance, low toxicity, cardiovascular protection and so on. Therefore, enalapril is an ideal chemotherapy sensitizer.

In summary, our finding shows that enalapril as an idea chemotherapy sensitizer can strongly restore chemosensitivity and significantly improves the efficacy of 5-FU in CRC with reduced toxicity of 5-FU. Our results have provided an experimental basis for the clinical application of enalapril in combination with 5-FU-based chemotherapeutics for CRC patients, especially for patients with poor chemotherapeutic tolerance and cardiovascular diseases. Considering the economic-effectiveness and low toxicity of the combination treatment, this research has a very important clinical value. Further clinical studies are necessary to confirm our findings in patients with CRC to precede a translation of our treatment strategy to clinical oncology.

## Materials and methods

### Human established and primary CRC cell lines and reagents

The established human CRC cell lines HCT116 and SW620 were obtained from American Type Culture Collection and human primary CRC cell lines P1, P2, P3, and P4 were freshly isolated from four different human colorectal tumors approved by the Ethics Committee of Zhongshan Hospital of Xiamen University and informed consent was obtained from all subjects. All cells cultured in the Dulbecco’s modified eagle medium supplemented with 10% fetal bovine serum (FBS), 100 units/mL penicillin, and 100 μg/mL streptomycin mycoplasma contamination. Cells were maintained in 5% CO_2_ incubator at 37 °C. Cell lines were routinely tested for mycoplasma and authenticated by short tandem repeat (STR) profiling. Enalapril and 5-FU were purchased from Sigma.

### Cell viability assay

Cells were treated with DMSO or enalapril (0–2000 μM) or (0–50 μM), or both agents together (100 μM enalapril + 10 μM 5-FU) for 48 or 72 h and cell viability was determined according to the MTT assay, as described in a previous study^46^.

### Detection of apoptosis

Cells were treated enalapril (100 μM), 5-FU (10 μM), or both agents together (100 μM enalapril + 10 μM 5-FU) for 48 h and Annexin V-fluoroisothiocyanate apoptosis detection kit was employed to determine the number of apoptotic cells by manufacturer protocols (Sigma), as described previously^46^.

### Western blot analysis

Cells were treated with enalapril (100 μM), 5-FU (10 μM), or both agents together (100 μM enalapril + 10 μM 5-FU) for 48 h and the whole-cell or nuclear extracts from CRC cells were prepared according to a standard protocol. Western blotting was performed as previously described^46^. The following antibodies were used: p65 (#sc-8008) and p-p65 (#sc-136548) (Santa Cruz Biotechnology, CA, USA), Cyclin D1 (#ab134175), VEGF-α (#ab46154), Bcl-2 (#ab196495), MMP-9 (#ab137867), MMP-2 (#ab92536), Ki67 (#ab92742), and c-Myc (#ab32072) (Abcam, Cambridge, UK), Cleaved Caspase-3 (##9661), p-STAT3(#9145), STAT3(#9139), XIAP(#2045), Snail(#3879), Vimentin(#5741), and E-cadherin(#14472) (Cell Signaling, MA, USA).

### Migration, invasion, and wound-healing assays

The migration and invasion capacities of CRC cells were evaluated through Transwell assay. In brief, the Transwell chambers were coated with or without 100 µl diluted Matrigel (Costar, Cambridge, MA). Then, cells were suspended within 100 µl serum-free medium and seeded into the upper Transwell chamber at a density of 2 × 10^5^ cells/ml, whereas the lower Transwell chamber was filled with medium containing 10% FBS. After treatment for 24 h in each group, cells on the upper chamber surface that had not migrated were carefully removed with a cotton swab. Afterwards, the remaining cells were fixed with cold 95% ethanol and stained with 0.1% crystal violet. The experiments were repeated in triplicate wells and the number of migrating cells was quantified by counting the cells in ten random fields per filter under the microscope. For wound-healing assays, cell monolayers were scratched with a pipette tip, and cell migration was evaluated after treated as described above for 24 h.

### Xenografts in nude mice

All animal experiments and procedures were carried out under the protocol approved by the Animal Care and Ethics Committee of Xiamen University. SW620 cells numbering 5 × 10^5^ (100 μl) were injected subcutaneously into the left rear flank of the 6- to 8-week-old female Balb/c nude mice (day 0). After 4 days of implantation when tumors had reached a palpable size, 24 mice bearing tumors were randomly divided into four groups (*n* = 6 in each group) and treated as follows: (a) control group (100 μl of 0.9% saline, oral gavage, daily); (b) enalapril group (0.6 mg/kg enalapril dissolved in saline, oral gavage, daily); (c) 5-FU group (30 mg/kg, intraperitoneal injection, twice weekly); and (d) 5-FU combined with enalapril group (30 mg/kg 5-FU, intraperitoneal injection, twice weekly; and 0.6 mg/kg enalapril dissolved in saline, oral gavage, daily). The animals were killed on day 24 and the tumors were excised. The tumor volume was calculated for three times a week according to the following formula: *V* = 1/2 (length × width^2^). The body weight change for each mouse was monitored every 4 days. Nude mice experiments were approved by the ethics committee of Xiamen University.

### Metastasis assay in mice

To evaluate metastatic capacity, SW620-FU cells (5 × 10^5^) were injected into the 6- to 8-week-old Balb/c female nude mice through the splenic vein or SW620-FU cells (1 × 10^6^) were injected into the cecal wall. Animals were anesthetized after 7–8 weeks to assess the metastatic capacity. Nude mice experiments were approved by the ethics committee of Xiamen University.

### Immunohistochemistry

Mice were killed and tumor samples were fixed with paraformaldehyde for immunohistochemical analysis as described previously^46^. Tissues were immunostained using antibodies against p65, p-p65, cyclin D1, VEGF-α, Bcl-2, MMP-9, MMP-2, Ki67, c-Myc, Cleaved Caspase-3, p-STAT3, STAT3, XIAP, Snail, Vimentin, and E-cadherin. For staining quantification, positive staining was quantified in ten visual fields at ×400 magnification from three tumors of each of the treatment group.

### Statistical analysis

Data are presented as mean ± SD and analyzed by Graph Prism 7.0 software. The significance of data was analyzed using the two-tailed Student’s *t*-test for parametric data and the Mann–Whitney test with Bonferroni corrections for non-parametric data. A difference of *P* < 0.05 was considered as statistically significant (*P < 0.05, **P < 0.01). All in vitro experiments were independently confirmed three times. The number of samples in each group ≥ 5 and statistical power analysis was used to ensure adequate sample size for detecting significant difference between samples. The variance is similar between groups that are being statistically compared.

## Supplementary information

Supplementary Figure Legends

Supplementary Figure 1

Supplementary Figure 2

Supplementary Figure 3

Supplementary Figure 4
