# Supplementary material for: Enalapril overcomes chemoresistance and potentiates antitumor efficacy of 5-FU in colorectal cancer by suppressing proliferation, angiogenesis, and NF-κB/STAT3-regulated proteins
Source: Cell Death Dis. 2020 Jun 24;11(6):477. doi: 10.1038/s41419-020-2675-x (PMC7314775; doi:10.1038/s41419-020-2675-x)
Supplement: Supplementary file 1 — Supplementary Figure Legends [file 41419_2020_2675_MOESM1_ESM.docx]

**Enalapril overcomes chemoresistance and potentiates antitumor efficacy of 5-FU in colorectal cancer by suppressing proliferation, angiogenesis, and** **NF-κB/STAT3-regulated proteins**

Yushan Yang^1,3,4,5^, Lulu Ma^1,3,4,5^, Yiming Xu^2,5^, Yun Liu^2^, Jinbo Fu^2^, and, Wenya Li^1,3,4^, Qiang Li^1,3,4^, Yifan Zhuang^1,3,4^, Lifeng Zhong^1,3,4^, Shuzhen Xu^1,3,4^, Jianchun Cai ^1,3,4^*, Yiyao Zhang ^1,3,4^*

^1^Departments of Gastrointestinal Surgery, Zhongshan Hospital of Xiamen University, Xiamen 361000, China

^2^General Surgery, Zhongshan Hospital of Xiamen University, Xiamen 361000, China.

^3^Gastrointestinal Oncology Center of Xiamen University, Xiamen 361000, China.

^4^Medical College of Xiamen University, Xiamen 361000, China

^5^These authors contributed equally to this work.

***Correspondence:** Yiyao zhang, Departments of Gastrointestinal Surgery, Zhongshan Hospital of Xiamen University, Xiamen 361000, China

**E-mail**: doctorzhyy@163.com

**Supplementary Figure Legends**

**Supplementary Figure 1. Enalapril overcomes 5-FU resistance in CRC cells in vitro.** (A) CRC cells were treated with indicated concentrations of enalapril (EP) or (B) 5-FU (FU) for 48 h, and the cell viability was measured by MTT assay. (C) HCT116 and SW620 cells were treated with EP (100 μM), FU (10 μM), or the two agents combined (E+F) for 24 h, and the cell viability was measured by MTT assay. The data are presented as means ± SD from three separate experiments (n=8 per group). Statistical analysis performed using two-tailed t-test (***P* < 0.01, **P* < 0.05).

**Supplementary Figure 2.** **Detection of cell cycle distribution and apoptosis by flow cytometry.**

(A) HCT116 cells were treated with EP (100 μM), FU (10 μM), or the two agents combined (E+F) for 48 h, the cell apoapsis was measured by flow cytometry using Annexin V/propidium iodide double staining.（B）HCT116 and SW620 cells were treated with EP (100 μM), FU (10 μM), or the two agents combined (E+F) for 48 h, the distribution of cell cycle was measured by flow cytometry. The data are presented as means ± SD from three separate experiments (n=6 per group). Statistical analysis performed using two-tailed t-test (***P* < 0.01, **P* < 0.05).

**Supplementary Figure 3. Combination of enalapril and 5-FU does not increase the toxic side effects in mice.**

**(A)** The body weight change for mice was evaluated every 4 days. **(B)** The final tumor weight of mice in each group was analyzed**. (C)** H&E staining was used to verify the liver change in the mice and the bar indicates 100 μm. **(D)** The levels of plasma blood urea nitrogen (BUN) and the levels of alanine aminotransferase (ALT) were analyzed at the end of the animal experiment. The data are presented as means ± SD (n=6 per group). Statistical analysis performed using two-tailed t-test (***P* < 0.01, **P* < 0.05).

**Supplementary Figure 4. The expression of total STAT3 and P65 in tumor tissue.**

(A) Immunohistochemical analysis of total STAT3 and P65 in tumor tissue samples treated with EP, FU, or the two agents combined (E+F). The bar indicates 100 μm. (B) The number of positive cells was quantified in 10 visual fields at 400× magnification. The data are presented as means ± SD from three separate experiments (n=6 per group). Statistical analysis performed using two-tailed t-test (***P* < 0.01, **P* < 0.05).
