# Supplementary figures and images for: Enalapril overcomes chemoresistance and potentiates antitumor efficacy of 5-FU in colorectal cancer by suppressing proliferation, angiogenesis, and NF-κB/STAT3-regulated proteins
Source: Cell Death Dis. 2020 Jun 24;11(6):477. doi: 10.1038/s41419-020-2675-x (PMC7314775; doi:10.1038/s41419-020-2675-x)

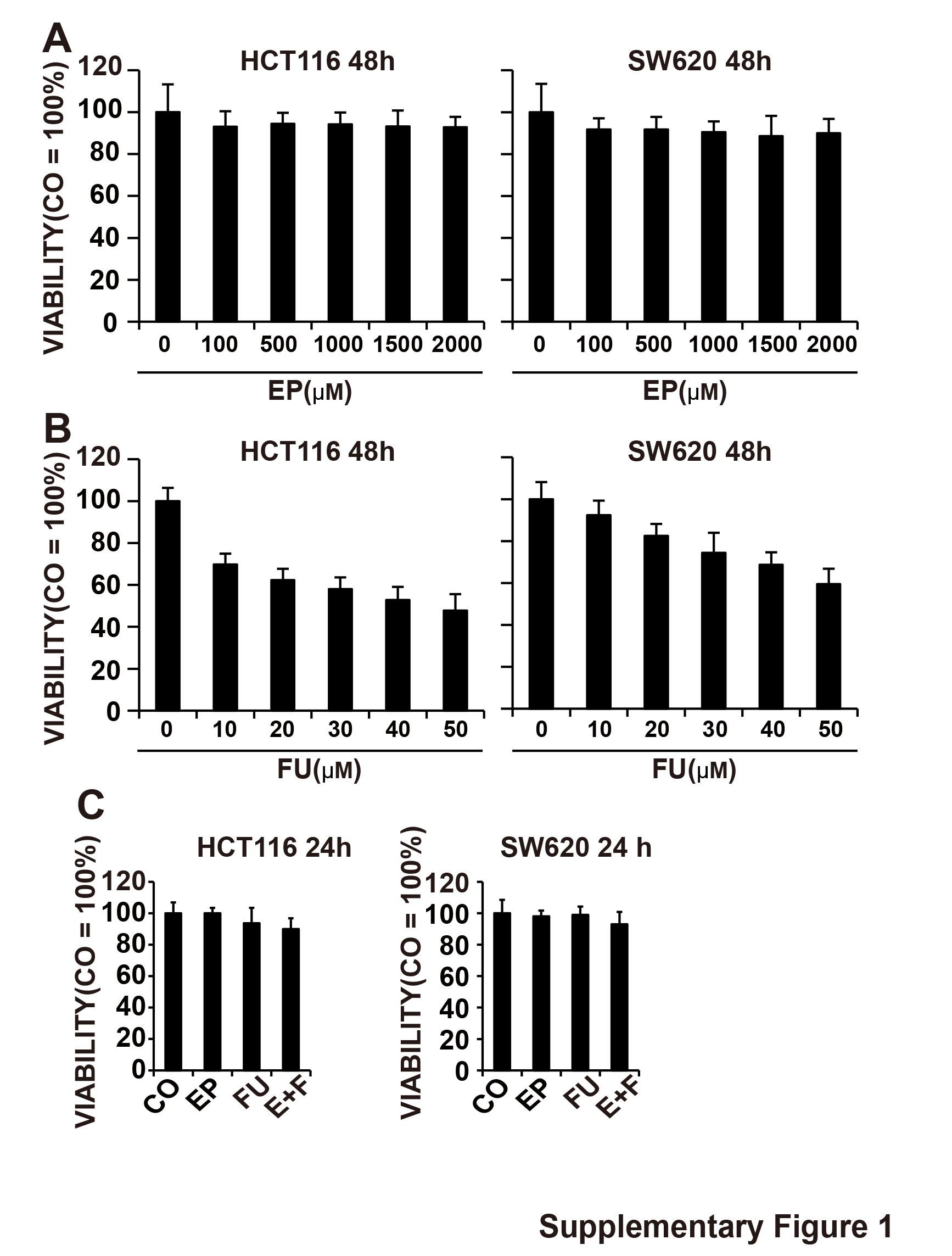

Supplement: Supplementary file 2 — Supplementary Figure 1 [file 41419_2020_2675_MOESM2_ESM.png]

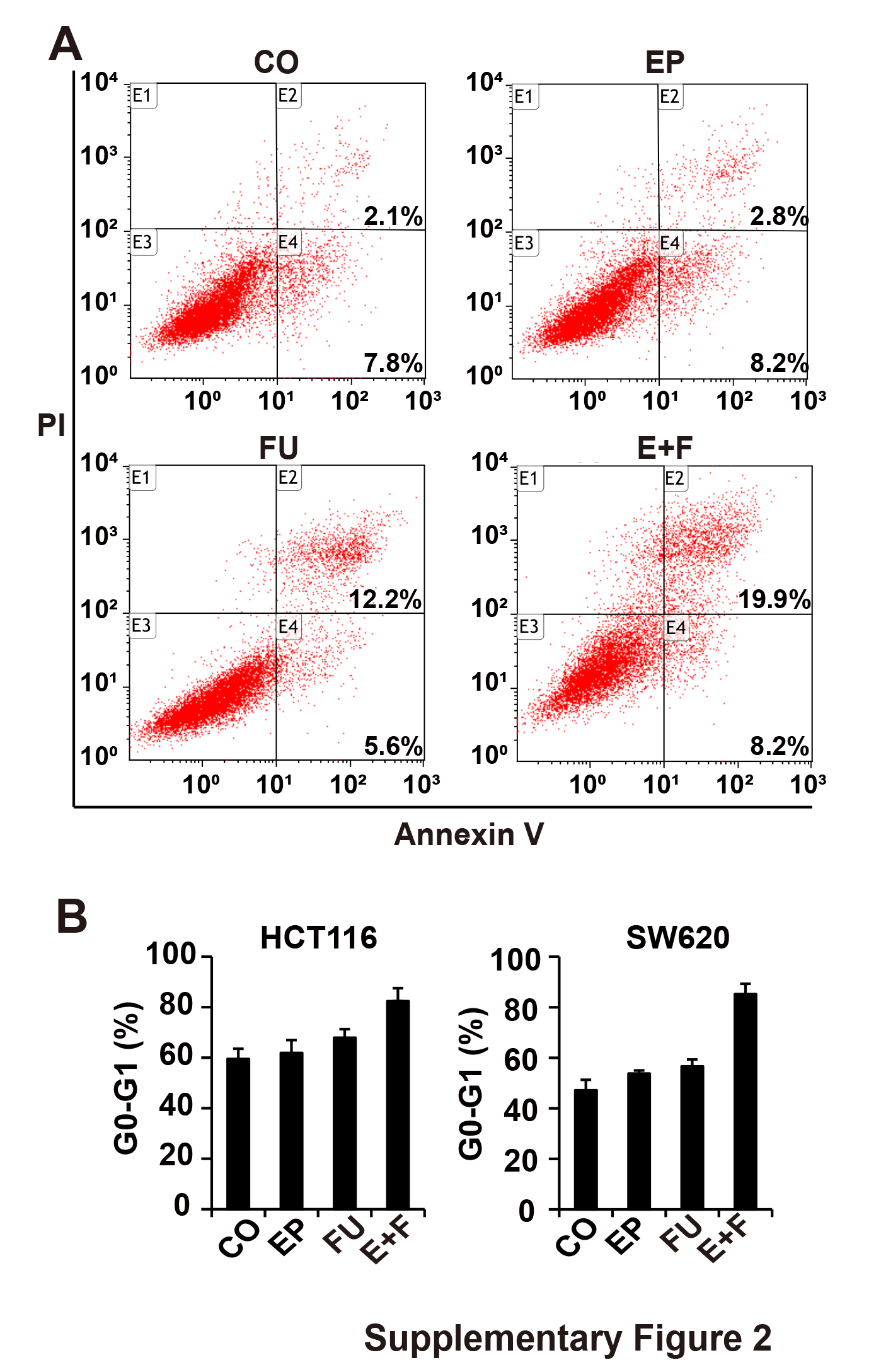

Supplement: Supplementary file 3 — Supplementary Figure 2 [file 41419_2020_2675_MOESM3_ESM.png]

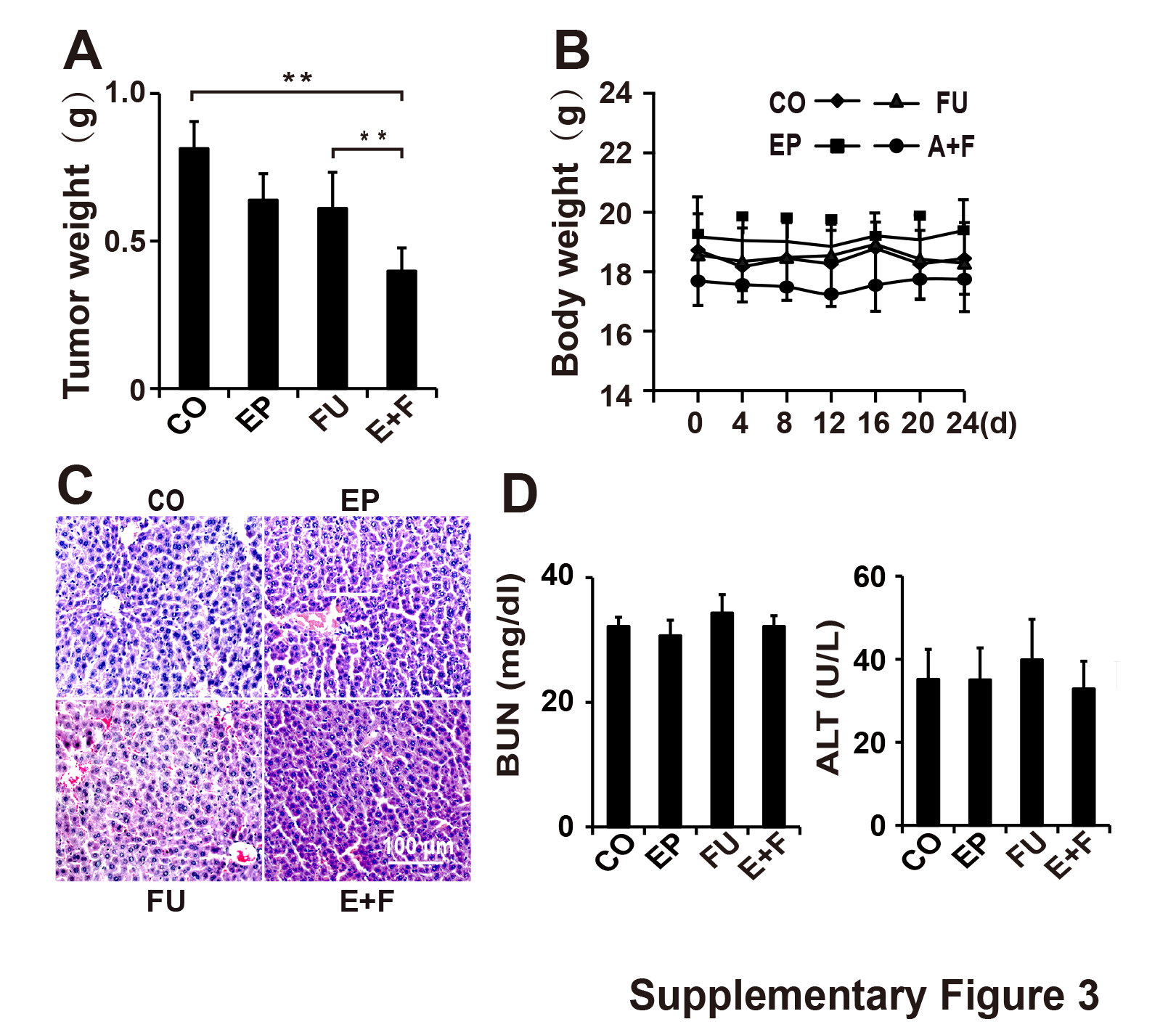

Supplement: Supplementary file 4 — Supplementary Figure 3 [file 41419_2020_2675_MOESM4_ESM.png]

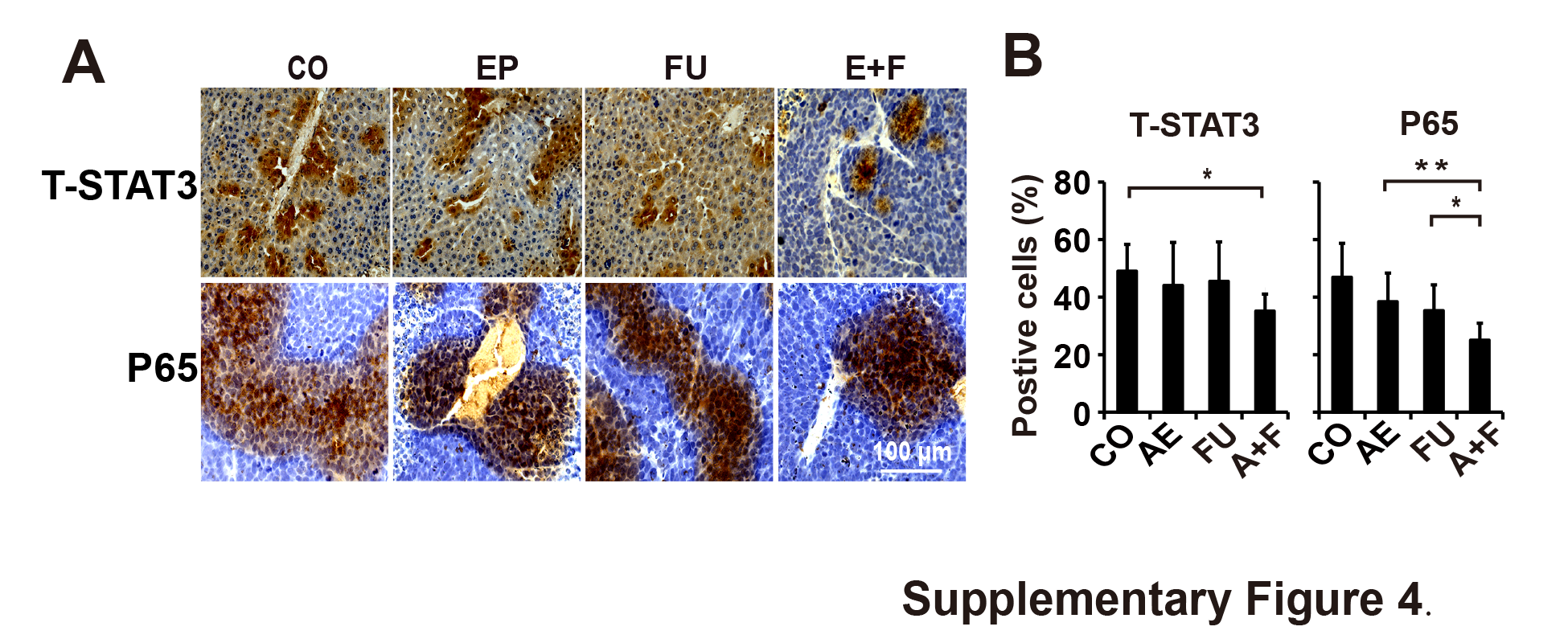

Supplement: Supplementary file 5 — Supplementary Figure 4 [file 41419_2020_2675_MOESM5_ESM.png]
